# Local inhibition of TGF-β1 signaling improves Th17/Treg balance but not joint pathology during experimental arthritis

**DOI:** 10.1038/s41598-022-07075-w

**Published:** 2022-02-24

**Authors:** Joyce Aarts, Arjan van Caam, Xinlai Chen, Renoud J. Marijnissen, Monique M. Helsen, Birgitte Walgreen, Elly L. Vitters, Fons A. van de Loo, Peter L. van Lent, Peter M. van der Kraan, Marije I. Koenders

**Affiliations:** Department of Experimental Rheumatology, Radboud Institute for Molecular Life Sciences, Radboud University Medical Center (Radboudumc), PO Box 9101, 6500 HB Nijmegen, The Netherlands

**Keywords:** Inflammation, Lymphocytes, Cytokines, Transforming growth factor beta

## Abstract

TGF-β1 is an important growth factor to promote the differentiation of T helper 17 (Th17) and regulatory T cells (Treg). The potential of TGF-β1 as therapeutic target in T cell-mediated diseases like rheumatoid arthritis (RA) is unclear. We investigated the effect of TGF-β1 inhibition on murine Th17 differentiation in vitro, on human RA synovial explants ex vivo, and on the development of experimental arthritis in vivo. Murine splenocytes were differentiated into Th17 cells, and the effect of the TGF-βRI inhibitor SB-505124 was studied. Synovial biopsies were cultured in the presence or absence of SB-505124. Experimental arthritis was induced in C57Bl6 mice and treated daily with SB-505124. Flow cytometry analysis was performed to measure different T cell subsets. Histological sections were analysed to determine joint inflammation and destruction. SB-505124 potently reduced murine Th17 differentiation by decreasing *Il17a* and *Rorc* gene expression and IL-17 protein production. SB-505124 significantly suppressed IL-6 production by synovial explants*.* In vivo, SB-505124 reduced Th17 numbers, while increased numbers of Tregs were observed. Despite this skewed Th17/Treg balance, SB-505124 treatment did not result in suppression of joint inflammation and destruction. Blocking TGF-β1 signalling suppresses Th17 differentiation and improves the Th17/Treg balance. However, local SB-505124 treatment does not suppress experimental arthritis.

## Introduction

Rheumatoid arthritis (RA) is an autoimmune disease of unknown etiology, that is characterized by chronic joint inflammation leading to destruction of articular cartilage and bone. CD4 + T cells are found in inflammatory infiltrates of the rheumatoid synovium and are known to play a central role in the development of RA^1–4^. In patients with RA, the T helper 17 (Th17) and regulatory T cells (Treg) balance is skewed in favor of Th17 cells, which contributes to the development of autoimmunity and inflammation^5^.

Transforming growth factor beta (TGF-β) is a growth factor that can differentiate CD4 + T cells into Th17 cells in the presence of IL-6 or IL-21^6^. TGF-β signaling and STAT3 activate and regulate expression of the transcription factor RAR-related orphan receptor C (*RORc*), the master regulator of Th17 cells^7,8^ . There are three isoforms of TGF-β: TGF-β1, TGF-β2, and TGF-β3^9^. Lymphoid cells produce mainly TGF-β1, which has a controversial role in inflammation and autoimmunity as it both suppresses and induces immune reactions^10^. Three canonical TGF-β receptors have been described: TGF-βRI, TGF-βRII, and the co-receptor TGF-βRIII, that together form the TGF-β receptor complex. When TGF-β binds to this receptor-complex, receptor-regulated Smad (R-Smad) proteins are phosphorylated and translocate to the nucleus to modulate target gene expression^11^.

In diseases such as cancer and atherosclerosis, the role of TGF-β1 is well defined, but its role in (experimental) arthritis is still not clear. TGF-β1 is a regulatory cytokine, having pleiotropic functions on immunity including promoting the expansion of Tregs^12^ and regulation of CD4+ T cell polarization^13^. TGF-β1 promotes the differentiation of Th17 as well as Treg cells, depending on the cytokine milieu^14^. This has resulted in quite some opposing phenotypes in mice transgenic for the TGF-β signaling pathway. On the one hand, mice with a T cell-specific deletion of TGF-βRII showed uncontrolled T cell activation and massive and fatal multi-organ inflammation^15^. On the other hand, mice with a deletion of the *Tgfb1* gene selectively in CD4+ and CD8+ T cells showed a defect in the generation of Th17 cells that protected them from experimental autoimmune encephalitis (EAE)^16^. In line with that the latter, mice subcutaneously injected with 100 μg anti-TGF-β1,2,3 antibody 1D11 failed to differentiate naïve CD4+ T cells into Th17 cells and were subsequently protected from EAE^17^.

Due to the dual role of TGF-β1 in regulating the immune system, the potential of targeting the TGF-β1 pathway as therapy for RA is unclear. In this study, we investigated the effect of inhibiting TGF-β1 signaling with the TGF-βRI inhibitor SB-505124 on murine Th17 differentiation in vitro, on cytokine production by human RA synovial explants ex vivo, and on the development of joint pathology in vivo during Th17-driven experimental arthritis.

## Materials and methods

### Patient donors

Synovial tissues from nine RA patients were obtained during joint replacement surgery from the orthopedics department of the Sint Maartenskliniek, Nijmegen, The Netherlands. Written informed consent was obtained from all patients. The study was approved by the local Ethics Review Board (CMO region Arnhem-Nijmegen, the Netherlands), approval number 2018-4319. All patients fulfilled the ACR/EULAR2010 RA classification criteria and were end-stage RA. The patient material was pseudonymized. Procedures were performed in accordance with the code of conduct for responsible use of human tissue in medical research. The presence of a synovial intimal lining was determined on 5 μm cryosections stained with hematoxylin and eosin (H&E) as previous described to confirm the synovial origin of the tissue^18^.

### Mice

Female C57BL/6N were purchased from Janvier-Elevage (Le Genest Saint Isle, France). Animals were used between 10 and 12 weeks of age. A standard diet and water were provided ad libitum. All animal procedures and experimental protocols were approved by the animal ethics committee of the Radboud University Nijmegen (permit RU-DEC 2018-0037). All procedures were performed according to the Institute of Laboratory Animal Research Guide for Laboratory Animals and were performed in compliance with the institutional and ARRIVE guidelines.

### Murine Th17 differentiation

T lymphocytes from spleens were isolated from naïve C57BL/6N mice as previously described^19^. 500,000 cells/well were cultured in a 24-wells plate for five days at 37 °C in an atmosphere of 5% CO_2_, in X-vivo medium (Lonza, LOT 8MB036) supplemented with 1% penicillin–streptomycin (Lonza 09-757F). Cells were activated with plate-bound anti-CD3 (5 µg/ml; BioLegend; clone 17A2) and soluble anti-CD28 (2.5 µg/ml; BioLegend; clone 37.51). After 2 h preincubation with or without 5 µM SB-505124 (Sigma-Aldrich), cells were differentiated into Th17 cells with αIL-2 (10 µg/ml; BioLegend; clone JES6-1A12), IL-1β (10 ng/ml; BioLegend), IL-6 (50 ng/ml; ITK diagnostics), IL-23 (10 ng/ml; ITK diagnostics) and increased concentrations of TGF-β1 (0.05, 0.5, 1 or 10 ng/ml, BioLegend). After five days of differentiation, the supernatant was collected for cytokine measurement and the cells were processed for RNA isolation (n = 3 biological replicates from one mouse) or flow cytometry (n = 3 mice).

### Luminex

Human and murine cytokines and chemokines (IL-17A, IL-10, TNF-α, GM-CSF, IFN-γ, IL-6 and IL-4) were measured by Luminex using BioPlex kits according to manufacturer’s instructions, Bio-Rad Laboratories) and analyzed using BioPlex Manager 4 software.

### Flow cytometry

Cells were stimulated for 4 h with phorbol myristate acetate (PMA; 50 ng/ml; Sigma-Aldrich), ionomycin (1 µg/ml; Sigma-Aldrich), and the Golgi-traffic inhibitor Brefeldin (1 µl/ml: BD Biosciences, Franklin Lakes, NJ, USA). Next, cells were stained with anti-CD3-FITC (145-2C11) (BioLegend), anti-CD4-PerCP/Cy5.5 (RM4-4) (BioLegend), fixable viability dye eFluor 780 (eBioscience), fixed and permeabilized using Cytofix/Cytoperm (BD Biosciences), followed by intra-cellular staining with anti-IL-17-PE (TC11-18H10.1) (BioLegend), anti-FOXP3-Alexa Fluor 647 (150D) (BioLegend) and anti-RORγt-BV421 (Q31-378) (BD Biosciences). The gating of T cells was as follows. First, cells were gated in an FSC-A vs. SSC-A dot plot, and subsequently gated in FSC-W vs. FSC-A and SSC-W vs. SSC-A dot plots to eliminate doublets. Living cells were gated based on FVD, and next CD3 + /CD4 + cells were gated on the basis of FMO controls. Finally, IL-17 + and FOXP3 + cells were determined in this gate as markers for Th17 and Treg cells, respectively. Cells were analyzed on the Cytoflex and subsequently using Kaluza software version 2.1.

### RNA isolation and quantitative real-time PCR

Cells from in vitro studies were collected in TRI Reagent for further RNA isolation as previously described^19^. Gene expression levels were determined by qPCR on the StepOnePlus sequence detection system (Applied Biosystems) using SYBR Green (Applied Biosystems) and 0.2 µM primers (Biolegio). GAPDH was used as reference genes. Relative quantification of the PCR signals was performed by comparing the cycle threshold (Ct) value of the gene of interest for each sample with the Ct values of the reference gene. Primer sequences were as follows (Table 1):

**Table 1:** List of murine oligonucleotide primer sequences.

Genes	5’–3’ forward	5’–3’ reverse
*Il17a*	caggacgcgcaaacatga	gcaacagcatcagagacacagat
*Rorc*	ctgtcctgggctaccctactga	aagggatcacttcaatttgtgttctc
*Il22*	ggtgcctttcctgaccaaac	cgtcaccgctgatgtgaca
*Gapdh*	ggcaaattcaacggcaca	gttagtggggtctcgctcctg

### Methylated bovine serum albumin/interleukin-1 (mBSA/IL-1) induced-arthritis

Acute inflammatory arthritis was induced in knee joints of C57BL/6N mice as previously described^20^ by intra-articular injection with 200 µg of mBSA (Sigma A-1009), followed by daily injections on days 0–2 with 250 ng of human IL-1β (BioLegend). At time of sacrifice, knee joints were isolated for histological analysis and qPCR. Draining popliteal and inguinal lymph nodes were isolated for flow cytometry analysis. The treatment with the TGF-βRI inhibitor SB-505124 (Sigma-Aldrich) was applied locally starting two hours before the induction of arthritis, and mice were injected intra-articularly on four consecutive days with either saline, 20% DMSO as vehicle control, or 75 nmol SB-505124 (Sigma-Aldrich) in 20% DMSO with an injection volume of 6 µl per joint. Anti-IL-17A antibodies (BioXCell) were used as positive control, administered as 50 µg antibody/mouse intra-peritoneally injected every other day (n = 8 mice/group). Mice were sacrificed by cervical dislocation four days after arthritis induction, and knee joints were subsequently isolated for histological analysis.

### Streptococcal cell wall (SCW) arthritis induction

Streptococcus pyogenes T12 organisms were cultured overnight in Todd-Hewitt broth. Cell wall fragments were prepared as described previously^21^. Arthritis was subsequently induced by injecting 25 µg SCW fragments into the knee joints of C57BL/6N mice (n = 7 per group), resulting in local, acute inflammation. Local SB-505124 (Sigma-Aldrich) treatment was applied as described above.

### Histology

Knee joints were processed for histological analysis and scored in line with the 'SMASH' recommendations for standardized microscopic arthritis scoring^22^. In short, isolated joints were fixed for at least four days in 4% formaldehyde, decalcified in 5% formic acid, and subsequently dehydrated and embedded in paraffin. Standard frontal sections of 7 µm were stained with H&E or Safranin O (SO) to study joint pathology. The severity of arthritis was scored on an arbitrary scale of 0–3, where 0 = no pathology and 3 = maximal pathology, for two different parameters (joint inflammation and cartilage proteoglycan (PG) depletion), on three semi-serial sections of the joint, spaced 140 µm apart, in a blindfolded manner.

### Immunohistochemistry

Protein expression of phosphorylated Smad2/3 was evaluated on 7 µm paraffin-embedded sections of murine synovial tissue (n = 3 per group), collected 1 h after (co-)injection of recombinant 10 ng TGF-β1 (Biolegend) with or without 75 nmol SB-505124 (Sigma-Aldrich) into the knee joints of naïve C57BL/6 N mice. For immunohistochemistry, endogenous peroxidase activity was blocked with 3% H_2_O_2_ (Merck Millipore) in methanol, and antigen retrieval was performed in 10 mM citrate buffer, pH 6.0 at 60 °C. Subsequently, sections were stained with primary antibodies: rabbit anti-mouse pSmad2/3 (Cell Signaling #3108) (1:300 for 60 min at RT), mouse or isotype. Subsequently, the sections were stained with biotinylated anti-mouse IgG H + L (1:100 for 30 min at RT). Next, a biotin-streptavidin detection system was used according to the manufacturer’s protocol (PK-6101; Vector Laboratories). Peroxidase was developed with diaminobenzidine (Sigma-Aldrich) and counterstained with hematoxylin for 60 s.

### Synovial explant culture

Synovial tissue was obtained during joint replacement surgery and tissue was freshly processed into standardized biopsies using a 3 mm biopsy punch (Stiefel) (n = 9 donors, mean of 2–6 biopsies per donor per condition). The synovial explants were subsequently cultured for 24 h at 37 °C and 5% CO_2_ in 200 µl X-vivo serum-free medium in the presence or absence of 5 µM SB-505124 (Sigma-Aldrich). After 24 h, culture medium was centrifuged 10 min, 241 × *g* to remove remaining cells. Before Luminex analysis, culture medium was centrifuged 10 min, 10.000 rpm.

### Statistical analysis

We assessed differences in the mean values of Th17 genes and proteins with and without 5 µM SB-505124 using the one-way ANOVA followed by Bonferroni post-test. We assessed differences in protein production by the human RA synovial explants with/without SB-505124 using a paired Student’s *t*-test. The differences in Th17 and Tregs levels between vehicle and SB-505124 injected mice were assessed using a Student *t*-test. Additionally, differences between PG depletion and inflammation were assessed by one-way ANOVA and Bonferroni’s multiple comparison test for all treatment groups.Statistical analysis and normality testing were performed using GraphPad Prism version 5.03 (GraphPad Software). *P*-values < 0.05 were considered significant.

### Ethics approval and consent to participate

Synovial tissue was obtained as remnant material from RA patients (n = 8) undergoing joint replacement surgery. Written informed consent was obtained from all patients. The study was approved by the local Ethics Review Board (SMK Nijmegen, The Netherlands, 2018-4319). Tissue was processed into standardized 6 mm biopsies as described previously^54^. Procedures were performed in accordance to the code of conduct for responsible use of human tissue in medical research.

### Consent for publication

All authors read the manuscript and gave their consent for publication.

## Results

### TGFB-RI inhibitor SB-505124 prevents TGF-β1-induced expression of Th17 genes and proteins

**Figure 1:**
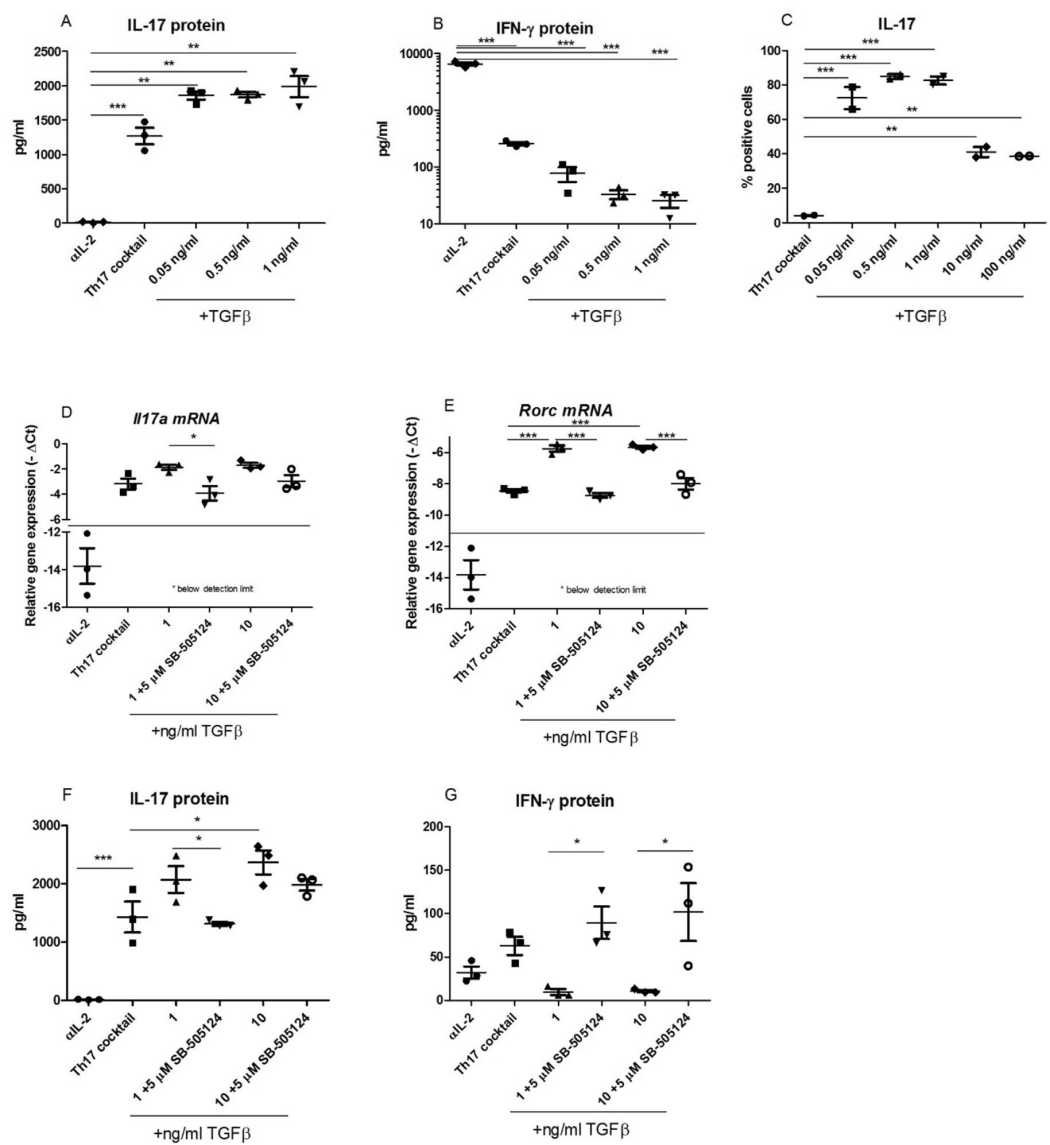
TGFB-RI inhibitor SB-505124 prevents TGF-β1-induced expression of Th17 genes and proteins. Splenocytes were differentiated into Th17 cells in the presence of αCD3 and αCD28 with either αIL-2 alone, Th17 cocktail (IL-1, IL-6, IL-23) with or without the addition of increased concentrations of TGF-β1 and SB-505124 (5 µM) for five days of culture. Cytokine levels in supernatant were measured using Luminex (**A,B,F,G**). CD4 + IL-17 + T cells cultured with increased concentrations of TGF-β1 were determined by flowcytometry (**C**). Gene expression was determined by QPCR (**D,E**). n = 3 mice/group (ABC) and n = 3 biological replicates from one mouse (DEFG), values are mean ± SEM. *P < 0.05, **P < 0.01, ***P < 0.001 calculated using one-way ANOVA followed by Bonferroni post-test.

To investigate the effect of inhibition of TGF-β1 signaling on murine Th17 differentiation in vitro*,* murine splenocytes were cultured with different concentrations of TGF-β1 in the presence or absence of 5 µM SB-505124. First, cells cultured with increasing concentrations of TGF-β1 showed a dose-dependent increase in IL-17 secretion (Fig. 1A) and a dose-dependent decrease in IFN-γ production (Fig. 1B). TGF-β1 also increased the percentage of Th17 cells (defined as CD4 + IL-17 + cells) when dosed 0.05 to 1 ng/ml (Fig. 1C). Remarkably, higher concentrations of 10 ng/ml and 100 ng/ml of TGF-β1 decreased the percentage of Th17 cells (Fig. 1C) without affecting the IL-17 production (Fig. 1F), demonstrating that IL-17 protein production not always correlates with Th17 differentiation levels. Higher concentrations of TGF-β1 did not affect the amount of apoptotic cells (Supplementary Fig. 1) but may inhibit proliferation as the absolute cell numbers were lower (Supplementary Fig. 2).

Addition of SB-505124 to these Th17 differentiation conditions supplemented with 1 ng/ml TGF-β1 resulted in significantly decreased *Il17a* mRNA expression, and in a trend for suppressed *Il17a* levels in the 10 ng/ml TGF-β1 group (Fig. 1D). Even more pronounced effects were observed for the lineage-specific transcription factor *Rorc* gene expression. In line with the data for *Il17a*, TGF-β1 significantly increased the expression of *Rorc* compared to the plain Th17 cocktail condition, and this TGF-β1-mediated effect could be completely counteracted by SB-505124 (Fig. 1E). Also at protein level, the inhibiting effect of SB-505124 on TGF-β1-mediated Th17 differentiation was observed. However, whereas the TGF-β1-induced increase in IL-17 production was completely and significantly abolished by 5 µM SB-505124 in the 1 ng/ml TGF-β1 group, the SB-505124-mediated suppression of IL-17 production in the 10 ng/ml TGF-β1 group is suboptimal with a dose of 5 µM, reaching only a trend of 30% reduction. The opposite of the TGF-β1- and SB-505124-induced effects on *Rorc* and IL-17 expression and production was observed on IFN-γ levels: the presence of TGF-β1 completely blocked IFN-γ production, which was reversed by addition of SB-505124 to the Th17 culture (Fig. 1F,G). These results indicate an important role for TGF-β1 during murine Th17 differentiation and suggest potency for SB-505124 to intervene in this process.

### Inhibiting TGF-β1 signaling using SB-505124 reduces IL-6 production by human RA synovial explants

**Figure 2:**
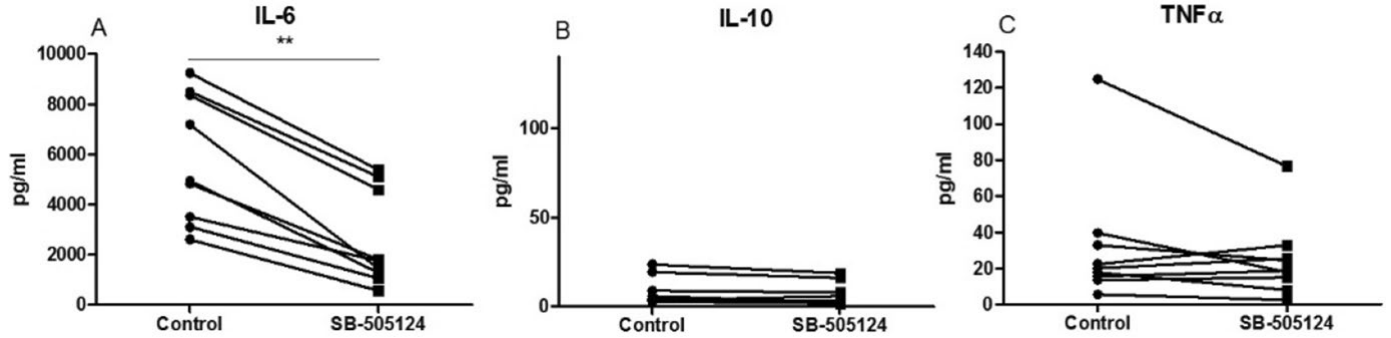
Inhibiting TGF-β1 signaling using SB-505124 significantly reduced IL-6 production by human RA synovial explants. Synovium biopsy punches were collected from synovium collected during joint replacement surgery. Explants were cultured for 24 h in the presence or absence of 5 µM SB-505124 and cytokine levels in the supernatants were measured by Luminex. Graphs show results for IL-6 **(A)**, IL-10 **(B)**, and TNFα **(C)**. N = 9 donors, mean of 2–6 biopsies per donor per condition. **P < 0.01, as determined by paired Student's *t*-test.

In addition to the observed effect of blocking TGF-β1 signaling by SB-505124 on murine Th17 differentiation, we investigated the potential of SB-505124 to inhibit the spontaneous production of inflammatory cytokines by human RA synovial explants. Interestingly, SB-505124 significantly inhibited the production of IL-6 (Fig. 2A) by the explants from 5802 pg/ml + /− 848 (mean + /− SEM) in the control group to 2565 pg/ml + /− 633 in the SB-505124 group. All our nine donor samples spontaneously produced high levels of IL-6 that was on average 56% inhibited in the presence of SB-505124, suggesting that active TGF-β1 signaling contributes to the inflammation in this synovial tissue. The levels of IL-10 were relatively low (Fig. 2B) and remained unaffected by SB-505124 treatment. For TNF-α production, not all donors responded similarly to the SB-505124 treatment. Five donors showed decreased levels, two donors increased levels and two donors showed no difference upon SB-505124 treatment (Fig. 2C). Other cytokines including IFN-γ, IL-4, GM-CSF, and IL-17 were not detectable.

### Intra-articular injection of SB-505124 does not induce joint inflammation and cartilage PG depletion in naïve mice

**Figure 3:**
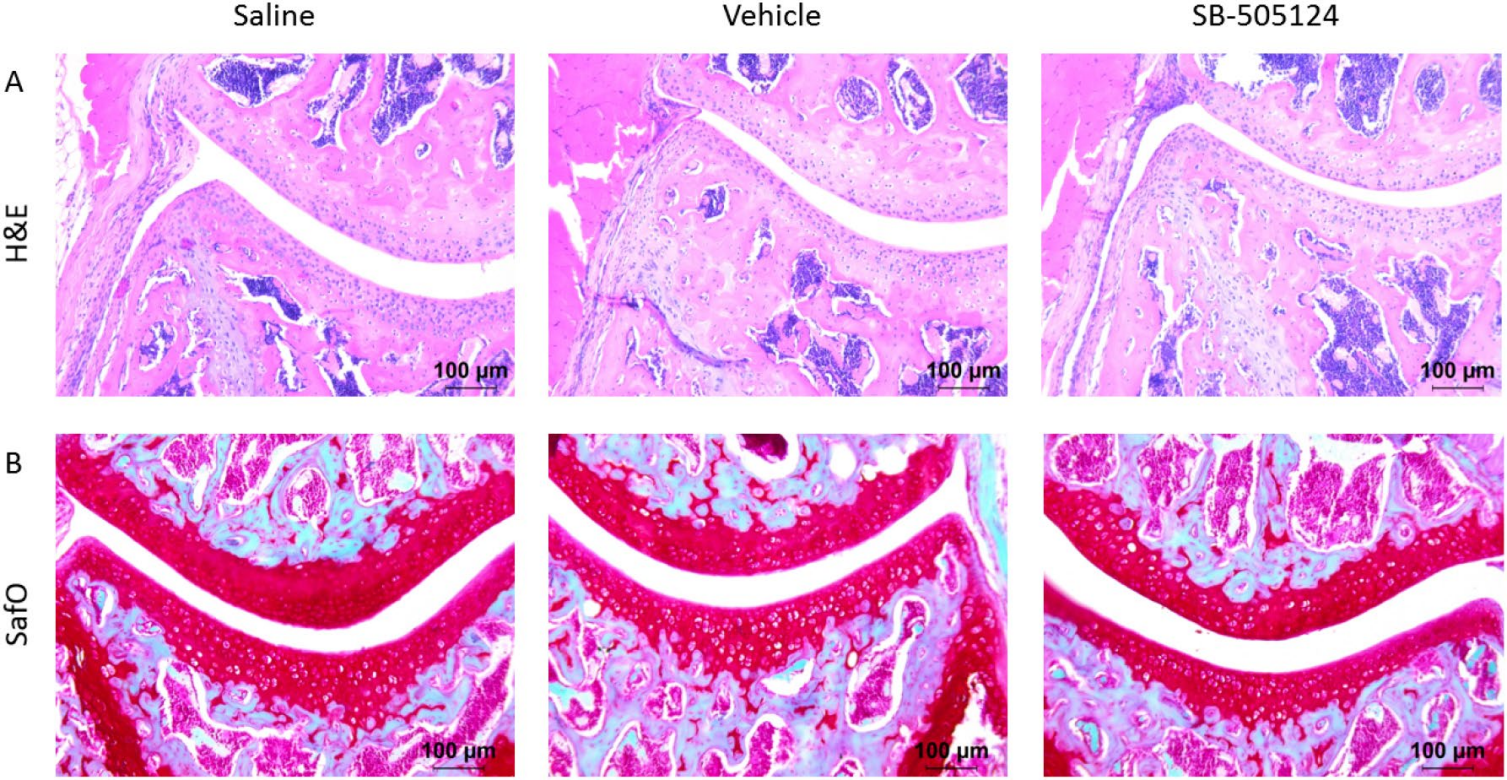
Intra-articular injection of SB-505124 does not induce joint inflammation and cartilage PG depletion. Mice were daily injected with 75 nmol SB-505124 or 20% DMSO as vehicle control from day 0–4, and sacrificed after 7 days. Knee joints were subsequently isolated for histological analysis of inflammation (HE stain, original magnification ×100) **(A)** and cartilage PG depletion (SafO stain, original magnification ×100). **(B)** Representative pictures of n = 4 joints per group.

Before inhibiting TGF-β1 signaling during experimental arthritis, we first aimed to study the potential joint pathology of local SB-505124 treatment in naïve mice, as TGF-β1 is not only important in T cell activation but also in cartilage homeostasis. Repeated intra-articular SB-505124 injections for four consecutive days did not induce synovial inflammation or cartilage proteoglycan depletion in naïve knee joints as observed on histology at day seven. Also the vehicle control 20% DMSO did not result in joint pathology, as these control mice showed a thin synovial lining and healthy cartilage similar to saline-injected knee joints (Fig. 3). Importantly, we demonstrated that our treatment regimen of daily intra-articular injections of SB-505124 can indeed inhibit local TGF-β1 signaling. By co-injection of recombinant TGF-β1 and the TGF-βRI inhibitor SB-505124 in naïve murine knee joints, we demonstrated that SB-505124 inhibits TGF-β1 activity in vivo, as indicated by reduced pSmad2/3 staining as marker for active TGF-β1 signaling by immunohistochemistry of synovium tissue of these mice (Supplementary Figs. 3 and 4).

### Intra-articular injection of SB-505124 enhances Tregs and suppresses Th17 cell levels in the draining lymph nodes of arthritic mice

**Figure 4:**
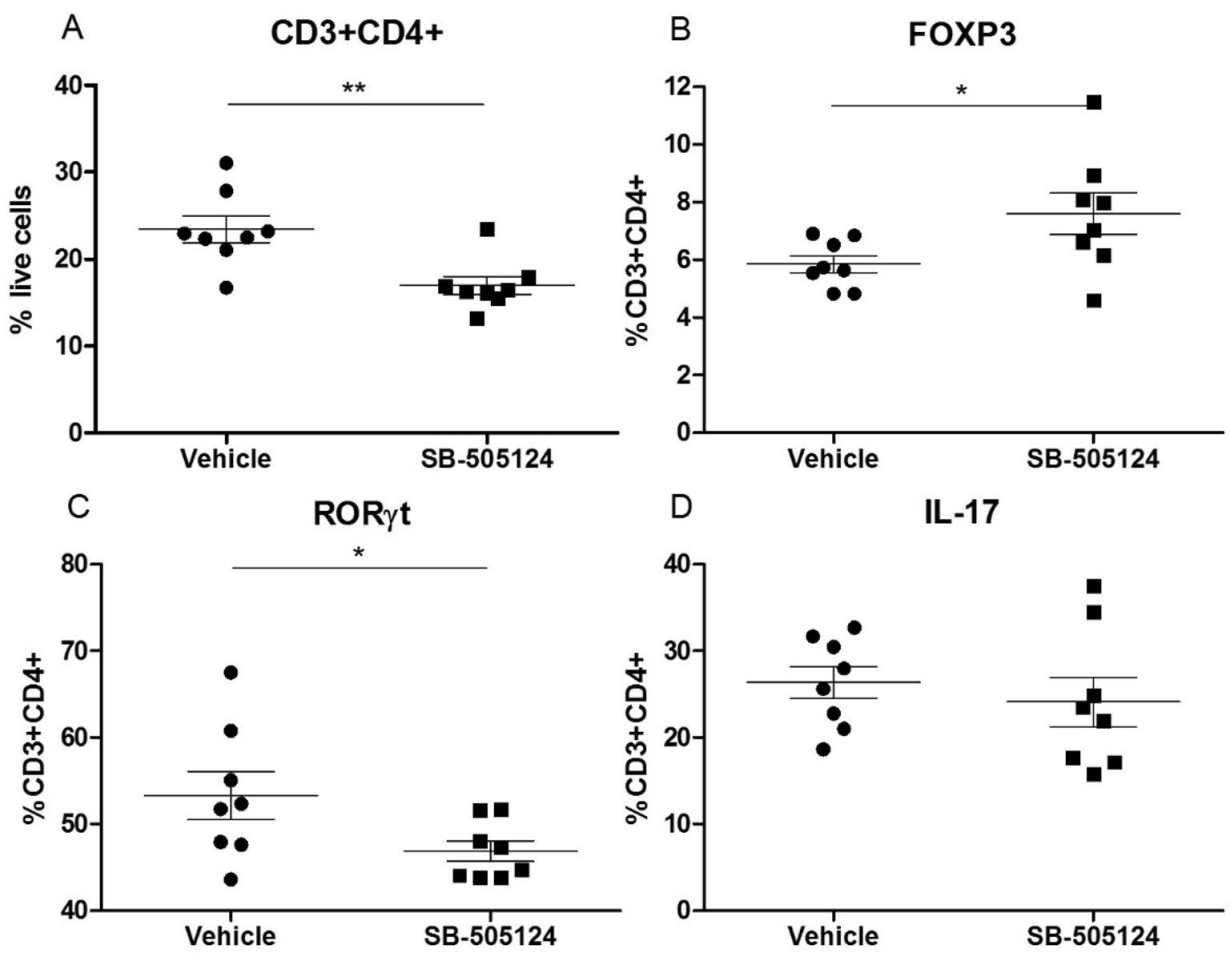
Intra-articular injection of SB-505124 enhances Tregs and suppresses Th17 cell levels in arthritic mice. Cells from draining lymph nodes (n = 8 mice/group) were stimulated for 4 h with PMA/ionomycin and analyzed by flow cytometry. The percentage of CD3 + CD4 + T cells was decreased in the SB-505124 injected mice **(A)**. Levels of Th17 cells were decreased by SB-505124 treatment **(B)**, whereas FOXP3 + T cells (Tregs) were increased. **(B)** Levels of IL-17 + T cells were similar in vehicle and SB-505124 injected mice. ** P < 0.01, * P < 0.05 by Student’s t-test. Values are mean +/- SEM.

To study the effect of inhibition of TGF-β1 signaling on Th17-driven experimental arthritis, we treated mice during IL-1/mBSA arthritis with daily intra-articular injections of SB-505124. Interestingly, in the draining lymph nodes of SB-505124-treated mice, we observed a significant reduction in CD3 + CD4 + T cells at day 4 (Fig. 4A). Moreover, we observed that inhibition of TGF-β1 signaling resulted in increased numbers of FOXP3 + CD3 + CD4 + T cells (Tregs) (Fig. 4B) and less RORγt + CD3 + CD4 + T cells (Fig. 4C), although IL-17 + CD3 + CD4 + cell numbers were not altered by local SB-505124 treatment (Fig. 4D). These results indicate that local treatment with SB-505124 affects T cell proliferation and Th17/Treg differentiation in this experimental arthritis model.

### Local inhibition of TGF-β1 signaling with SB-505124 does not decrease arthritis joint pathology

**Figure 5:**
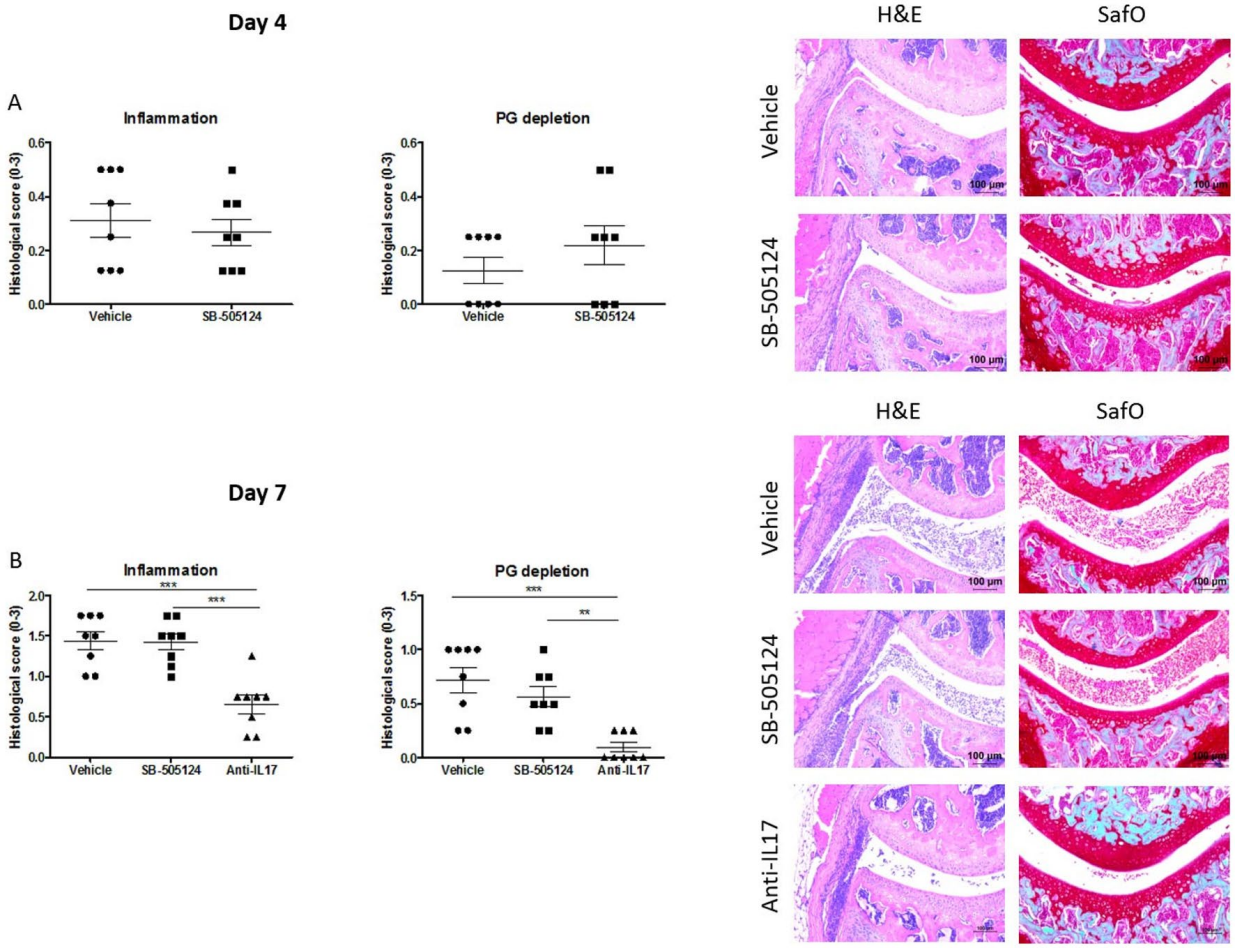
Intra-articular injections of SB-505124 do not decrease joint inflammation and PG depletion during experimental arthritis. Mice were daily injected i.a. with vehicle or SB-505124 for four days. Total knee joints were isolated for histopathologic analysis (n = 8 mice/group). Joint inflammation (H&E stain, original magnification ×100) and PG depletion (Safranin O staining, original magnification ×100) on day 4 **(A)** and day 7 **(B)** were analyzed on histological slides. Values are mean ± SEM. ** P < 0.01, *** P < 0.001 by one-way ANOVA and Bonferroni’s multiple comparison test **(B)** for all treatment groups.

By subsequent histological analysis, the effect of local inhibition of TGF-β1 signaling on arthritis severity was studied in more detail. Despite the skewed Th17/Treg balance in the draining lymph nodes of these arthritic mice, no differences were observed on joint inflammation and cartilage PG depletion on day four (Fig. 5A) and day seven (Fig. 5B) of arthritis between the SB-505124 group and its vehicle control. As expected, the positive control treatment using anti-IL-17 antibodies significantly reduced arthritis pathology (Fig. 5B). Additionally, no effects were observed in T cell-independent SCW arthritis after local SB-505124 treatment (supplementary Fig. 5).

## Discussion

RA patients may benefit from the inhibition of the Th17 pathway, either by neutralizing the main effector cytokine IL-17^23^, or by targeting further upstream via its transcription factor RORγt^24^ or Th17-inducing cytokines^25,26^. In this study, we explored the potential of targeting TGF-β1 signaling to inhibit Th17 cells and suppress Th17-driven arthritis, this in view of the importance of this growth factor in the differentiation of these cells.

Recently, it was shown that newly diagnosed as well as DMARD-treated RA patients showed significant increased plasma levels of TGF-β1 compared to healthy controls. However, the role of TGF-β1 in the disease process is complex, and it is therefore unclear whether the TGF-β1 pathway can be used for therapeutic targeting^27^. Previous studies in experimental arthritis models showed contradictory results with pro- or anti-inflammatory roles for TGF-β1. For instance, recombinant TGF-β1 injections in naïve rat and mouse joints induced joint inflammation with synovial infiltration of T lymphocytes and neutrophils^28–30^. Treatment of arthritic rats with monoclonal antibody 1D11.16 (anti-TGF-β1/2/3 antibody) inhibited inflammation and bone resorption^31^. Additionally, intraperitoneally (IP) injected HTS466284 (TGF-β type 1 receptor kinase inhibitor) prevented experimental arthritis in mice^32^. IP treatment with the specific TGF-β1 blocking peptide p17 reduced severity of CIA, however these effects were not statistically significant^33^.

In contrast, other studies showed that IP administration of TGF-β1 reduced the incidence and severity of CIA, especially when injected in late disease^34,35^. In rats with SCW-induced arthritis, IP^36^ and intramuscular injection^37^ of TGF-β1 at peak of inflammation suppressed the development of arthritis, whereas anti-TGF-β1/2/3 in CIA mice increased pro-inflammatory cytokines and arthritis severity^38,39^. Furthermore, an increase in TGF-β1, TGF-β2, and TGF-β3 during the remission phase of CIA suggests an important role for TGF-β in regulating the disease^40^. These opposing findings indicate a differential role for TGF-β, probably depending on the type of animal model, concentration, route of administration, disease stage, and site of inflammation.

In our in vitro studies, we observed that blocking TGF-β1 during Th17 differentiation efficiently and significantly reduced *Rorc* mRNA expression, but not IL-17 protein production. TGF-β1 is important in T cell differentiation, but also in dampening of the immune response of various T and B lymphocytes^41^. TGF-β1 inhibits differentiation and activation of specific T helper subsets by suppressing their lineage-specific transcription factors such as T-Bet and GATA-3, which are critical for Th1 and Th2 responses, respectively^42^. The importance of TGF-β in regulating T cell responses in vivo has been strengthened by the observation that mice lacking TGF-βRII specifically on T cells develop lethal multi-organ inflammation^15^. Also TGF-β1−/− mice develop multi-organ inflammation due to an impaired control of T cell activation and differentiation^43^. The phenotype of the TGF-β1−/− mice is completely rescued if mice are crossed to an MHCII knockout background, highlighting a crucial role for TGF-β1 in regulating pathological CD4 + T cell responses^44^. Furthermore, subsequent studies using mice with T cell-specific deletions of Smad2 and Smad3 showed that intracellular signaling via Smad2/3 is essential for the TGF-β1-mediated inhibition of effector T cells^45^. In our in vivo experiment, intra-articular SB-505124 treatment during experimental arthritis caused a clear reduction in Th17 levels. However, this did not result in suppression of arthritis, suggesting that (1) the Th17 pathway is not that important in this model, (2) TGF-βRI is redundant and TGF-βRII/III and Smad-independent signaling continues, (3) the remaining (Th17) cells are highly active due to loss of TGF-β1-mediated dampening, or (4) that the treatment with SB-505124 had a too limited duration in time on TGF-β1 signaling to suppress the arthritis measured at end stage of our in vivo experiment. Unfortunately, our study design did not enable us to investigate the activation of the local Th17 cells and other immune cells by checking cytokine levels in the joints. However, this would be in line with our in vitro studies where we observed that blocking TGF-β1 during Th17 differentiation efficiently and significantly reduced the *Rorc* mRNA expression but not the IL-17 production. From the potent effects of the anti-IL-17 treatment group as reference control, we can conclude that IL-17 is an important cytokine to block during this phase of this arthritis model.

TGF-β1 is often considered as an immunosuppressive cytokine, inhibiting for instance TNF-α, IFN-γ and IL-1β^10^. Interestingly, when inhibiting the TGF-β1 signaling pathway by SB-505124 on human RA synovium explants, IL-6 protein production by RA synovium was significantly reduced, suggesting that TGF-β1 is an important inducer of IL-6 in arthritic synovium. Several studies indicate the potential crosstalk and importance of the IL-6/TGF-β signaling pathways in various cell types. In line with our observation, we have previously shown that TGF-β1 stimulates the production of IL-6 by primary articular chondrocytes^46^ and by the chondrocyte G6 cell line, but also downregulates the IL-6 receptor^46^. TGF-β1 also stimulates IL-6 production in PBMCs^47^. Furthermore, TGF-β1 down-regulates IL-6 signaling in intestinal epithelial cells^48^, showing that TGF-β1 regulation of IL-6 signaling is cell type, tissue and context dependent. Another study showed that TGF-β can synergize with IL-6 in T cells by promoting the degradation of FOXP3 leading to more Th17 differentiation (22977658). In our ex vivo studies, synovial explants are originating from end-stage RA patients undergoing total knee or hip replacement, and thereby less active in secreting cytokines than early RA tissue. However, even with this less inflamed tissue, we observed an interesting suppression of the proinflammatory cytokine IL-6 in all donors after inhibition of TGF-β1 signaling by SB-505124.

In our approach we chose to use the small molecule inhibitor SB-505124 to inhibit TGF-β1 signaling, which has an advantage over antibodies in tissue and cell penetration^49,50^. Disadvantage is the short half-life^51^, therefore SB-505124 must be administered frequently. With daily i.a. injections, we observed that this compound highly efficiently blocked TGF-β1-mediated Smad2/3 phosphorylation (supplementary Figs. 3 and 4), without SB-505124 directly affecting the cartilage. However, as TGF-β1 is essential for normal cartilage homeostasis, a longer follow-up or treatment would have been required if SB-505124 had been successful in reducing arthritis pathology.

By SB-505124 injections, we specifically inhibited the TGF-βRI-Smad signaling pathway during T cell differentiation in vivo. We did not identify the exact mechanism in our study, but literature provides some interesting insights to hypothesize on this. During the mBSA/IL-1-induced arthritis model, macrophage-derived cytokines like IL-6, but also IL-2, play an essential role^52^. This environment is ideal for Th17 development and therefore this model is highly IL-17-dependent. However, this IL-6-dependent Th17 differentiation was blocked by SB-505124, potentially leaving the way open for T cells to develop into Treg cells in the presence of IL-2. This may have resulted in the improved Th17/Treg balance during experimental arthritis.

In our proof-of-concept study to demonstrate the potential of controlling Th17 cells by targeting TGF-β signalling, we included as positive control anti-IL17 treatment, since the arthritis model we used is known to be dependent on CD4 + T cells and IL-17^20,53^. Depletion of CD4 + T lymphocytes in mBSA/IL-1-induced arthritis led to dose-dependent T cell proliferation in draining lymph nodes in response to mBSA. Anti–IL17 antibody administered during our mBSA/IL-1-induced arthritis markedly reduced disease, confirming that the model is indeed IL-17 dependent^20,53^. However, our blocking of TGF-β1 signalling apparently did not sufficiently suppress the formation or activity of Th17 cells, as arthritis pathology was not affected by local SB-505124 treatment, showing that our strategy to target the Th17 pathway upstream is not as effective as blocking the main effector cytokine IL-17 itself.

## Conclusions

We revealed suppressive effects of SB-505124 on Th17 differentiation in vitro and on the Th17/Treg balance in arthritic mice. However, local SB-505124 treatment did not suppress joint inflammation and destruction during experimental arthritis. This indicates that, despite the importance of TGF-β1 in Th17 differentiation, inhibiting TGF-β1 signalling is therefore not an adequate way to target Th17-driven inflammation.

## Supplementary Information

Supplementary Figures.

## Data Availability

All data generated or analyzed during this study are included in this published article (and its supplementary information files).

## Abbreviations

EAE: Experimental autoimmune encephalitis
H&E: Hematoxylin and eosin
PG: Cartilage proteoglycan
RA: Rheumatoid arthritis
RORc: RAR-related orphan receptor C
SCW: Streptococcal cell wall
Th17: T helper 17 cell
T reg: Regulatory T cell
TGF-β: Transforming growth factor beta

## References

[1] Cope AP, Schulze-Koops H, Aringer M (2007). The central role of T cells in rheumatoid arthritis. Clin. Exp. Rheumatol..

[2] Cope AP (2008). T cells in rheumatoid arthritis. Arthritis Res. Ther..

[3] Panayi GS, Lanchbury JS, Kingsley GH (1992). The importance of the T cell in initiating and maintaining the chronic synovitis of rheumatoid arthritis. Arthritis Rheum..

[4] Van Boxel JA, Paget SA (1975). Predominantly T-cell infiltrate in rheumatoid synovial membranes. N. Engl. J. Med..

[5] Wang W (2012). The Th17/Treg imbalance and cytokine environment in peripheral blood of patients with rheumatoid arthritis. Rheumatol. Int..

[6] Zhang S (2018). The role of transforming growth factor beta in T helper 17 differentiation. Immunology.

[7] Laurence A (2007). Interleukin-2 signaling via STAT5 constrains T helper 17 cell generation. Immunity.

[8] Yang XO (2007). STAT3 regulates cytokine-mediated generation of inflammatory helper T cells. J. Biol. Chem..

[9] Yamamoto T (1996). Expression of transforming growth factor-beta isoforms in human glomerular diseases. Kidney Int..

[10] Gonzalo-Gil E, Galindo-Izquierdo M (2014). Role of transforming growth factor-beta (TGF) beta in the physiopathology of rheumatoid arthritis. Reumatol. Clin..

[11] Itoh Y, Saitoh M, Miyazawa K (2017). Smad3-STAT3 crosstalk in pathophysiological contexts. Acta Biochim. Biophys. Sin. Shanghai..

[12] Robertson SA (2009). Seminal fluid drives expansion of the CD4+CD25+ T regulatory cell pool and induces tolerance to paternal alloantigens in mice. Biol. Reprod..

[13] Chen W, Ten Dijke P (2016). Immunoregulation by members of the TGFbeta superfamily. Nat. Rev. Immunol..

[14] Yoshida Y (2014). The transcription factor IRF8 activates integrin-mediated TGF-beta signaling and promotes neuroinflammation. Immunity.

[15] Marie JC, Liggitt D, Rudensky AY (2006). Cellular mechanisms of fatal early-onset autoimmunity in mice with the T cell-specific targeting of transforming growth factor-beta receptor. Immunity.

[16] Li MO, Wan YY, Flavell RA (2007). T cell-produced transforming growth factor-beta1 controls T cell tolerance and regulates Th1- and Th17-cell differentiation. Immunity.

[17] Veldhoen M, Hocking RJ, Flavell RA, Stockinger B (2006). Signals mediated by transforming growth factor-beta initiate autoimmune encephalomyelitis, but chronic inflammation is needed to sustain disease. Nat. Immunol..

[18] Humby F (2019). Synovial cellular and molecular signatures stratify clinical response to csDMARD therapy and predict radiographic progression in early rheumatoid arthritis patients. Ann. Rheum. Dis..

[19] Marijnissen RJ (2011). Increased expression of interleukin-22 by synovial Th17 cells during late stages of murine experimental arthritis is controlled by interleukin-1 and enhances bone degradation. Arthritis Rheum..

[20] Lawlor KE, Campbell IK, O'Donnell K, Wu L, Wicks IP (2001). Molecular and cellular mediators of interleukin-1-dependent acute inflammatory arthritis. Arthritis Rheum..

[21] van den Broek MF, van den Berg WB, van de Putte LB, Severijnen AJ (1988). Streptococcal cell wall-induced arthritis and flare-up reaction in mice induced by homologous or heterologous cell walls. Am. J. Pathol..

[22] Hayer S (2021). 'SMASH' recommendations for standardised microscopic arthritis scoring of histological sections from inflammatory arthritis animal models. Ann. Rheum. Dis..

[23] Kellner H (2013). Targeting interleukin-17 in patients with active rheumatoid arthritis: Rationale and clinical potential. Ther. Adv. Musculoskelet. Dis..

[24] Chang MR, Lyda B, Kamenecka TM, Griffin PR (2014). Pharmacologic repression of retinoic acid receptor-related orphan nuclear receptor gamma is therapeutic in the collagen-induced arthritis experimental model. Arthritis Rheumatol..

[25] Biggioggero M, Crotti C, Becciolini A, Favalli EG (2019). Tocilizumab in the treatment of rheumatoid arthritis: An evidence-based review and patient selection. Drug Des. Dev. Ther..

[26] Yamagata T, Skepner J, Yang J (2015). Targeting Th17 effector cytokines for the treatment of autoimmune diseases. Arch. Immunol. Ther. Exp. (Warsz).

[27] Samimi Z (2019). The impaired gene expression of adenosine monophosphate-activated kinase (AMPK), a key metabolic enzyme in leukocytes of newly diagnosed rheumatoid arthritis patients. Mol. Biol. Rep..

[28] Allen JB (1990). Rapid onset synovial inflammation and hyperplasia induced by transforming growth factor beta. J. Exp. Med..

[29] Fava RA (1991). Transforming growth factor beta 1 (TGF-beta 1) induced neutrophil recruitment to synovial tissues: Implications for TGF-beta-driven synovial inflammation and hyperplasia. J. Exp. Med..

[30] van Beuningen HM, van der Kraan PM, Arntz OJ, van den Berg WB (1994). Transforming growth factor-beta 1 stimulates articular chondrocyte proteoglycan synthesis and induces osteophyte formation in the murine knee joint. Lab Invest..

[31] Wahl SM, Allen JB, Costa GL, Wong HL, Dasch JR (1993). Reversal of acute and chronic synovial inflammation by anti-transforming growth factor beta. J. Exp. Med..

[32] Sakuma M (2007). TGF-beta type I receptor kinase inhibitor down-regulates rheumatoid synoviocytes and prevents the arthritis induced by type II collagen antibody. Int. Immunol..

[33] Gonzalo-Gil E (2013). Transforming growth factor (TGF)-beta signalling is increased in rheumatoid synovium but TGF-beta blockade does not modify experimental arthritis. Clin. Exp. Immunol..

[34] Kuruvilla AP (1991). Protective effect of transforming growth factor beta 1 on experimental autoimmune diseases in mice. Proc. Natl. Acad. Sci. U S A.

[35] Santambrogio L, Hochwald GM, Leu CH, Thorbecke GJ (1993). Antagonistic effects of endogenous and exogenous TGF-beta and TNF on auto-immune diseases in mice. Immunopharmacol. Immunotoxicol..

[36] Brandes ME, Allen JB, Ogawa Y, Wahl SM (1991). Transforming growth factor beta 1 suppresses acute and chronic arthritis in experimental animals. J. Clin. Invest..

[37] Song XY, Gu M, Jin WW, Klinman DM, Wahl SM (1998). Plasmid DNA encoding transforming growth factor-beta1 suppresses chronic disease in a streptococcal cell wall-induced arthritis model. J. Clin. Invest..

[38] Sancho D (2003). CD69 downregulates autoimmune reactivity through active transforming growth factor-beta production in collagen-induced arthritis. J. Clin. Invest..

[39] Thorbecke GJ (1992). Involvement of endogenous tumor necrosis factor alpha and transforming growth factor beta during induction of collagen type II arthritis in mice. Proc. Natl. Acad. Sci. U S A.

[40] Marinova-Mutafchieva L, Gabay C, Funa K, Williams RO (2006). Remission of collagen-induced arthritis is associated with high levels of transforming growth factor-beta expression in the joint. Clin. Exp. Immunol..

[41] Li MO, Wan YY, Sanjabi S, Robertson AK, Flavell RA (2006). Transforming growth factor-beta regulation of immune responses. Annu. Rev. Immunol..

[42] Li MO, Sanjabi S, Flavell RA (2006). Transforming growth factor-beta controls development, homeostasis, and tolerance of T cells by regulatory T cell-dependent and -independent mechanisms. Immunity.

[43] Kulkarni AB (1993). Transforming growth factor beta 1 null mutation in mice causes excessive inflammatory response and early death. Proc. Natl. Acad. Sci. U S A.

[44] Letterio JJ (1996). Autoimmunity associated with TGF-beta1-deficiency in mice is dependent on MHC class II antigen expression. J. Clin. Invest..

[45] Gu AD, Wang Y, Lin L, Zhang SS, Wan YY (2012). Requirements of transcription factor Smad-dependent and -independent TGF-beta signaling to control discrete T-cell functions. Proc. Natl. Acad. Sci. U S A.

[46] Wiegertjes R (2019). TGF-beta dampens IL-6 signaling in articular chondrocytes by decreasing IL-6 receptor expression. Osteoarthr. Cartil..

[47] Turner M, Chantry D, Feldmann M (1990). Transforming growth factor beta induces the production of interleukin 6 by human peripheral blood mononuclear cells. Cytokine.

[48] Walia B, Wang L, Merlin D, Sitaraman SV (2003). TGF-beta down-regulates IL-6 signaling in intestinal epithelial cells: Critical role of SMAD-2. FASEB J..

[49] Khera N, Rajput S (2017). Therapeutic potential of small molecule inhibitors. J. Cell Biochem..

[50] Zhong S, Jeong JH, Chen Z, Chen Z, Luo JL (2020). Targeting tumor microenvironment by small-molecule inhibitors. Transl. Oncol..

[51] Sierra JR, Cepero V, Giordano S (2010). Molecular mechanisms of acquired resistance to tyrosine kinase targeted therapy. Mol. Cancer.

[52] Lawlor KE, Wong PK, Campbell IK, van Rooijen N, Wicks IP (2005). Acute CD4+ T lymphocyte-dependent interleukin-1-driven arthritis selectively requires interleukin-2 and interleukin-4, joint macrophages, granulocyte-macrophage colony-stimulating factor, interleukin-6, and leukemia inhibitory factor. Arthritis Rheum..

[53] Egan PJ, van Nieuwenhuijze A, Campbell IK, Wicks IP (2008). Promotion of the local differentiation of murine Th17 cells by synovial macrophages during acute inflammatory arthritis. Arthritis Rheum..

[54] Broeren MG (2016). Functional tissue analysis reveals successful cryopreservation of human osteoarthritic synovium. PLoS ONE.

