# Supplementary material for: Local inhibition of TGF-β1 signaling improves Th17/Treg balance but not joint pathology during experimental arthritis
Source: Sci Rep. 2022 Feb 24;12:3182. doi: 10.1038/s41598-022-07075-w (PMC8873460; doi:10.1038/s41598-022-07075-w)
Supplement: Supplementary file 1 — Supplementary Figures. [file 41598_2022_7075_MOESM1_ESM.docx]

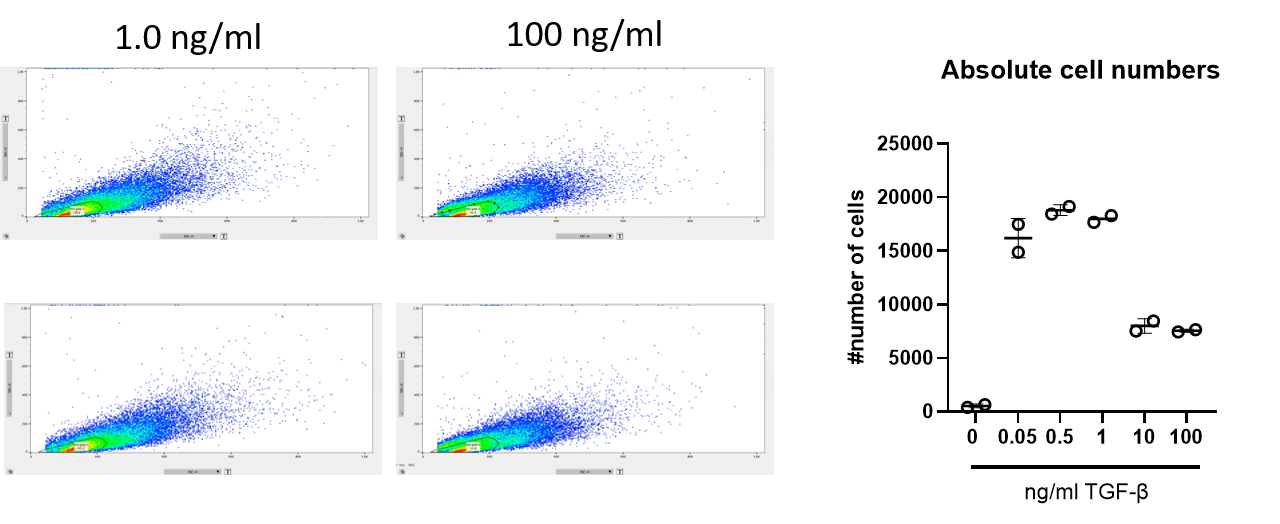


**Supplementary figure S1.** **Similar forward and side scatter plots with 1.0 ng/ml and 100 ng/ml TGF-β1.** Splenocytes were differentiated into Th17 cells in the presence of αCD3 and αCD28 with either αIL-2 alone, Th17 cocktail (IL-1, IL-6, IL-23) with or without the addition of increasing concentrations of TGF-β1. Cells were determined by flow cytometry.

*
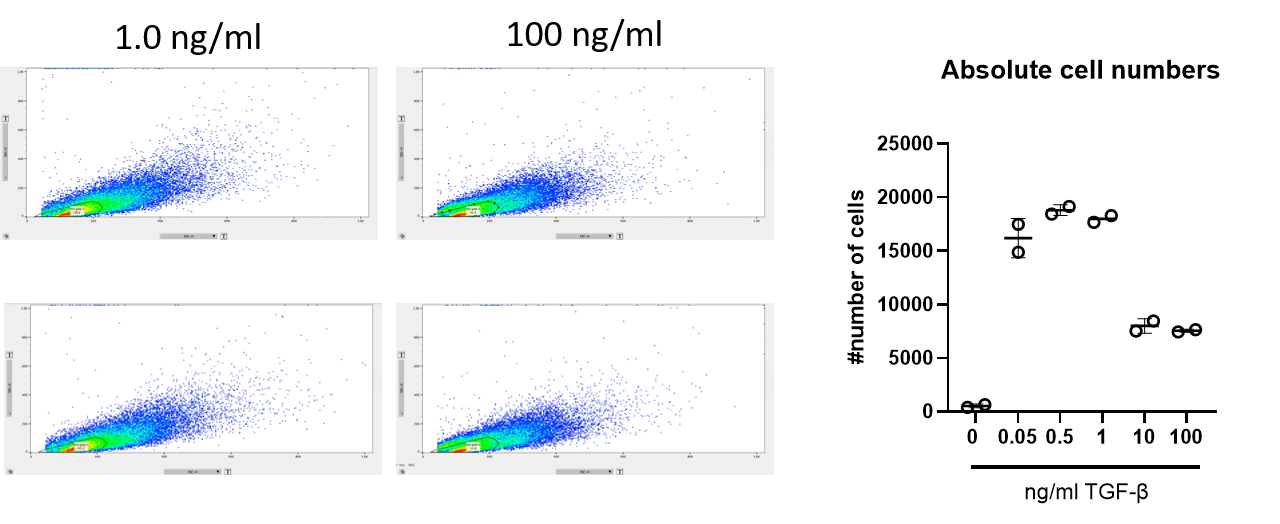
*

**Supplementary figure S2. Higher concentrations of TGF-β decrease absolute cell numbers of CD4+ IL-17+ T cells.** Splenocytes into Th17 cells in the presence of αCD3 and αCD28 with either αIL-2 alone, Th17 cocktail (IL-1, IL-6, IL-23) with or without the addition of increasing concentrations of TGF-β1. Cells were determined by flow cytometry.

DMSO + TGFβ SB-505124 + TGFβ

A B


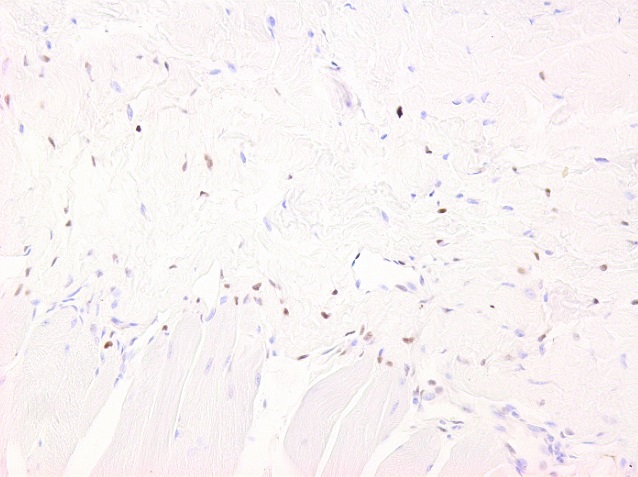

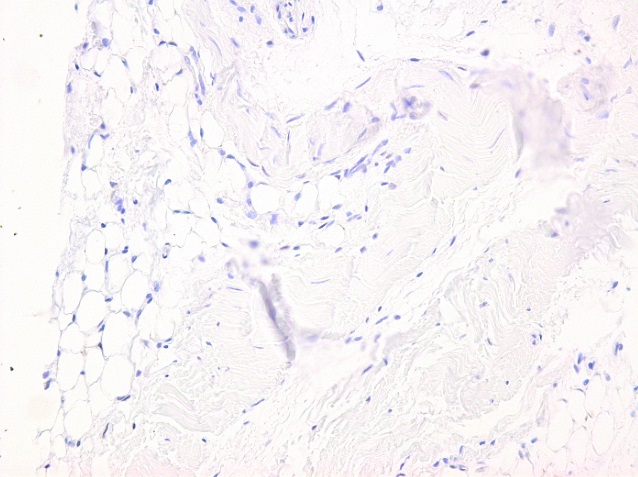


**Supplementary figure S3. Intra-articular injection of SB-505124 + TGFβ results in less pSMAD2/3 confirming the efficacy of SB-505124.** Synovium from naïve mice was collected to determine pSMAD2/3 3 hours after intra articular injection of SB-505124 with TGFβ . Injection with SB-505124 (B) resulted in less pSMAD2/3 compared to TGFβ injected mice (A) (n=3 mice).

DMSO + TGFβ SB-505124 + TGFβ

A B


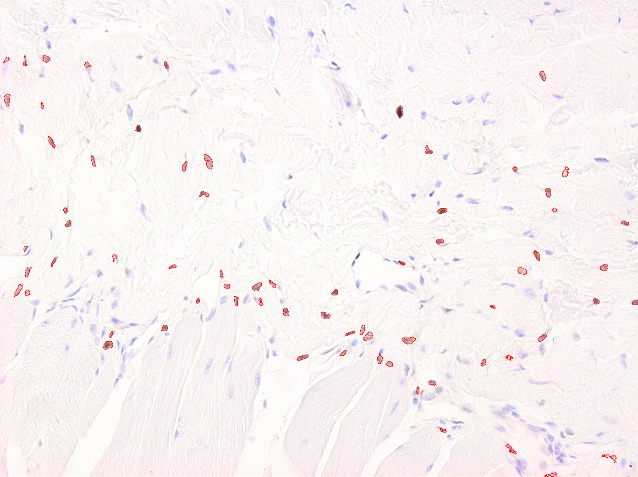

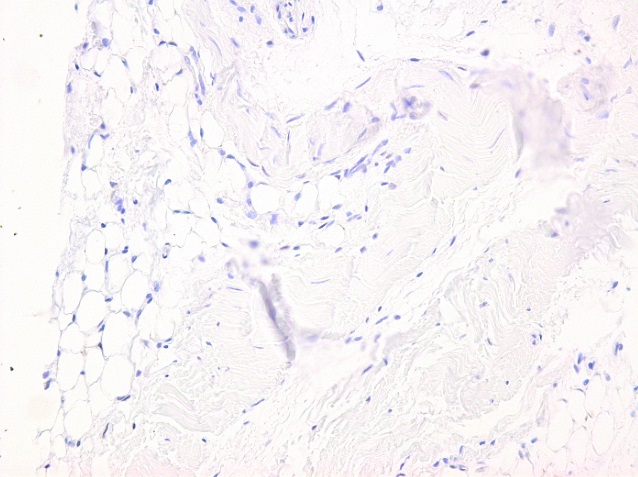


**Supplementary figure S4. Intra-articular injection of SB-505124 + TGFβ results in less pSMAD2/3 confirming the efficacy of SB-505124.** Synovium from naïve mice was collected to determine pSMAD2/3 3 hours after intra articular injection of SB-505124 with TGFβ . Injection with SB-505124 (B) resulted in less pSMAD2/3 compared to TGFβ injected mice (positive cells highlighted with use of ImageJ) (A) (n=3 mice).


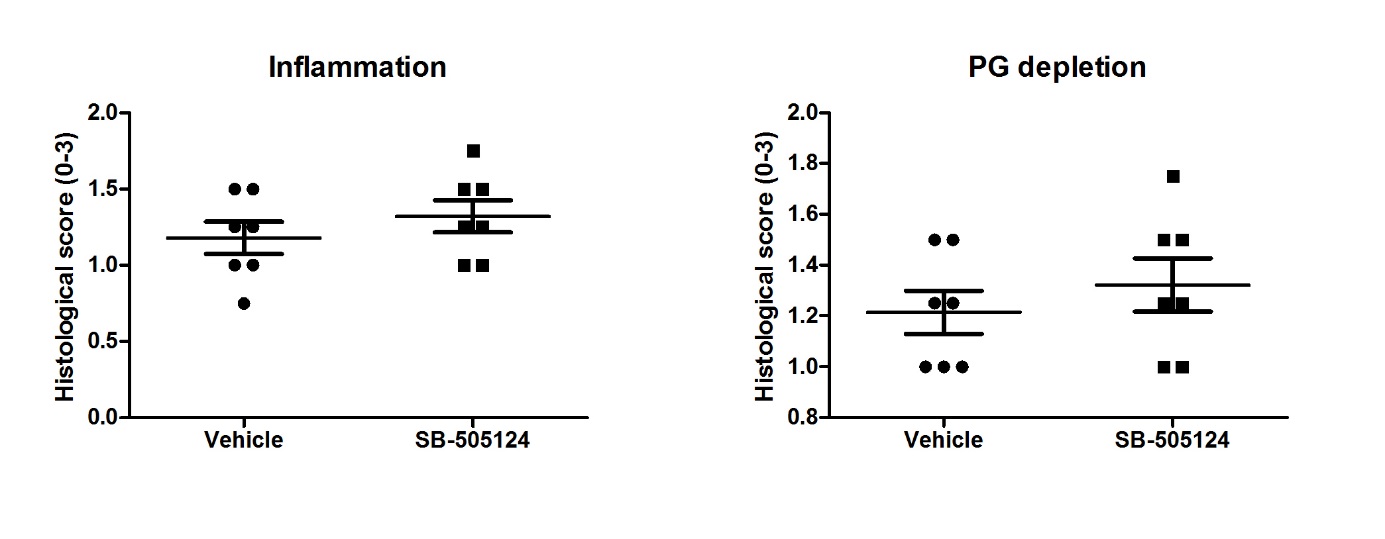
A B

**Supplementary figure S5.** **Repeated intra-articular injections of SB-505124 do not decrease joint inflammation and cartilage PG depletion during streptococcal cell wall (SCW) arthritis.** Mice were daily injected i.a. with vehicle or SB-505124 for 4 days. Total knee joints were isolated for histopathologic analysis (n=7 mice/group). Joint inflammation (H&E stain, original magnification 100x) (A) and PG depletion (Safranin O staining, original magnification 100x) (B) on day 4 were analyzed on histological slides. Values are mean ±SEM.
